# Epidemiological characteristics of comorbidities in childhood Attention-Deficit/Hyperactivity Disorder and common comorbidity patterns

**DOI:** 10.3389/fpubh.2026.1838844

**Published:** 2026-07-21

**Authors:** Xiaolu Ji, Haojie Meng, Qianqi Liu, Yang Li, Xu Wang

**Affiliations:** 1 Department of Pediatrics, The Affiliated Hospital of Yangzhou University, Yangzhou, Jiangsu, China; 2 Department of Children Health Care, Children's Hospital of Nanjing Medical University, Nanjing, Jiangsu, China; 3 Department of Neurology, Children's Hospital of Nanjing Medical University, Nanjing, Jiangsu, China; 4 Clinical Medical Research Center, Children's Hospital of Nanjing Medical University, Nanjing, Jiangsu, China

**Keywords:** Attention-Deficit/Hyperactivity Disorder, childhood, comorbidity, principal component analysis, systematic cluster analysis

## Abstract

**Objective:**

This study aims to explore the epidemiological features of medical and psychological comorbidities among children with Attention-Deficit/Hyperactivity Disorder (ADHD) and analyze the comorbidity profiles. The findings are expected to enhance clinical recognition of comorbid conditions and provide evidence for early screening and intervention.

**Methods:**

A retrospective analysis was performed on children diagnosed with ADHD at Children's Hospital of Nanjing Medical University between December 2015 and January 2025. Comorbidity was operationalized as ADHD accompanied by at least one other chronic condition or health problem in this study. The 10 most prevalent chronic conditions and health problems were defined based on parental medical history, on-site physical examinations, and blood sample testing. Systematic cluster analysis and principal component analysis (PCA) were employed to explore the comorbidity patterns in children with ADHD.

**Results:**

A total of 52,098 children diagnosed withs ADHD were enrolled in this study, including 41,124 males, accounting for 78.9% of the total study population. The age distribution showed that 92.6% of the children were aged 6–11 years. Among all enrolled subjects, 10,797 children (20.7%) had comorbid chronic conditions or health problems. Tic disorder was the most common comorbid condition. Regarding comorbidity combinations, the most frequent one was the co-occurrence of intellectual disability and autism spectrum disorder (ASD), with a total of 189 cases (*n* = 189, 25.7%). PCA identified a core comorbidity pattern for ADHD: tic disorders, intellectual disabilities, epilepsy, sleep problems, and obesity.

**Conclusion:**

Comorbid conditions are not rare among pediatric ADHD populations. The burden imposed by comorbidity in children with ADHD is substantial and thus cannot be neglected in clinical management. Based on the comorbidity patterns identified in this study, comprehensive clinical assessment covering both ADHD and its potential comorbidities, as well as integrated, individualized intervention strategies, are essential to optimize the long-term outcomes and quality of life for children with ADHD.

## 1 Introduction

Attention-Deficit/Hyperactivity Disorder (ADHD) is a common neurodevelopmental disorder characterized by core symptoms of inattention, hyperactivity, and impulsivity. In school-age children, the prevalence of ADHD is estimated at 5.3% based on population-representative epidemiological samples (1). ADHD symptoms typically manifest more prominently in school-aged children, are more common in boys, and tend to persist into adulthood (2). Children with ADHD frequently co-occur with mood disorders, anxiety disorders, and conduct disorders (3). As research advances, it has become increasingly evident that ADHD co-occurring with tic disorders, language development disorders, and autism spectrum disorder (ASD) are also not uncommon. These comorbid conditions often exacerbate social, emotional, and psychological impairments in affected children, thereby increasing the disease burden (4).

Although numerous studies have explored comorbidities associated with ADHD, reliable epidemiological data and distribution patterns for various comorbid conditions remain scarce. Previous research has predominantly focused on exploring the associations between ADHD and specific comorbidities, with relatively limited descriptions involving multiple concurrent comorbidities (5, 6).

This study aims to evaluate the epidemiological characteristics and comorbidity patterns of ADHD in children at our center. The findings of this study are expected to enhance clinicians' understanding of ADHD comorbidities, facilitate early detection of these comorbid conditions, and promote the development of targeted early intervention strategies, ultimately improving the clinical management and long-term outcomes of children with ADHD.

## 2 Methods

### 2.1 Data sources

**Figure 1:**
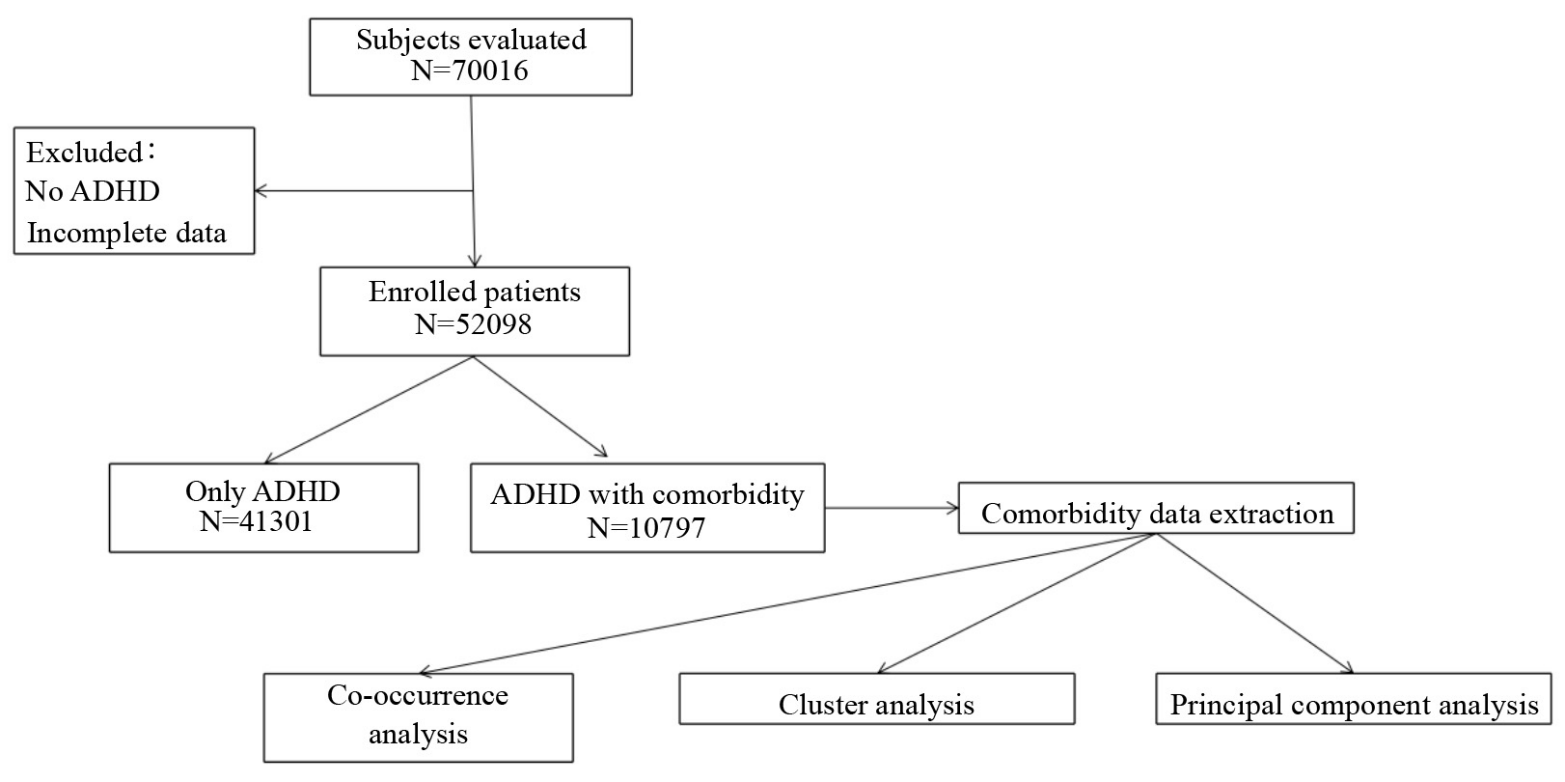
Flowchart of patient enrollment and comorbidity analysis in children with ADHD.

This study was approved by the Ethics Committee of Children's Hospital of Nanjing Medical University. Clinical trial registration: not applicable. Retrospective collection and analysis were performed on clinical data of children diagnosed with ADHD in our hospital from December 2015 to January 2025. A total of 52,098 children and adolescents aged 6–17 years clinically diagnosed with ADHD according to standardized diagnostic criteria were enrolled in this study, with no restriction on gender. Those with incomplete demographic information or missing key clinical data were excluded from the analysis. ADHD was diagnosed strictly in accordance with the DSM-5 diagnostic criteria, through standardized parental behavioral rating questionnaires (Conners' Child Behavior Checklist, CBCL; Swanson, Nolan, and Pelham-IV, SNAP-IV), structured clinical interviews (the ADHD module of the Diagnostic Interview Schedule for Children-IV, DISC-IV) conducted by developmental and behavioral pediatricians, and comprehensive assessment of functional impairment (Weiss Functional Impairment Rating Scale, WFIRS). The enrollment process of patients and the analysis of comorbidities are shown in Figure 1.

### 2.2 Comorbidity

Comorbidity was defined as the simultaneous presence of two or more chronic conditions or health problems in the study subjects. Specifically, in this study, it referred to ADHD accompanied by at least one additional chronic medical or psychological disorder. 10 chronic conditions or health problems were included in the comorbidity analysis, namely tic disorders, ASD, articulation disorders, learning disabilities, mood disorders, vitamin deficiencies, obesity, intellectual disabilities, epilepsy, and sleep problems. The 10 comorbidities analyzed in the present study were not all comorbid conditions in total. Instead, they were selected as the 10 most prevalent clinical comorbidities in children with ADHD for subsequent analysis. The diagnostic criteria for each condition were standardized as follows: tic disorders, and ASD were diagnosed in accordance with DSM-5 diagnostic criteria; articulation disorders were based on ICD-11 and ICF-CY diagnostic criteria for children with articulation disorders and related conditions; learning disabilities were diagnosed in line with DSM-5 criteria; mood disorders were diagnosed according to CCMD-3 criteria; vitamin deficiency referred to deficiency of vitamin A or D, defined as serum levels below the lower reference limit for age-matched peers; obesity was defined as a BMI ≥ P95th percentile relative to children of the same age and sex; intellectual disability followed DSM-5 diagnostic criteria; epilepsy was diagnosed based on the guidelines issued by the International League Against Epilepsy; sleep problems were defined as the presence of symptoms including difficulty falling asleep, frequent nighttime awakenings, early morning awakening, or excessive daytime sleepiness persisting for at least 3 months. All ADHD comorbidities in this study were determined via a unified clinical diagnostic workflow. According to the children's clinical manifestations on admission, routine physical examination and laboratory blood tests were performed to screen for suspected comorbidities. Clinical interviews were conducted to collect developmental, behavioral, emotional and medical historical information provided by parents, based on which clinicians raised initial suspicion of potential comorbid conditions.

Children with suspected comorbidities received further standardized developmental, behavioral and psychological assessments. Evaluations and definitive diagnoses were made by physicians with intermediate or higher professional titles from the departments of Developmental and Behavioral Pediatrics, Pediatric Neurology, and Child Psychiatry and Psychology. All diagnostic procedures were strictly performed in accordance with the corresponding diagnostic criteria. Meanwhile, we retrospectively reviewed multiple historical medical records of enrolled participants and extracted previously confirmed comorbid diagnoses for subsequent statistical analysis.

### 2.3 Research methods

All data processing procedures and statistical analyses employed in this study were implemented using R programming language. Multiple R packages were utilized to complete the analysis.

#### 2.3.1 Demographic correlation analysis

Independent samples *t*-tests and chi-square tests were employed to compare differences in gender and age distribution between two groups of children with ADHD: those with comorbidities and those without comorbidities. A two-tailed *p*-value < 0.05 was considered statistically significant in all analyses.

#### 2.3.2 Co-occurrence analysis

For cases with comorbidities, the co-occurrence patterns of comorbid conditions in children with ADHD were comprehensively evaluated using absolute co-occurrence frequency and the Jaccard similarity coefficient. Statistical significance was assessed using Fisher's exact test and BH correction.

#### 2.3.3 Cluster analysis

This study employed individual-level hierarchical cluster analysis to explore patterns of multiple coexisting diseases. First, the diagnostic information for 10 chronic diseases among study subjects was converted into binary codes (assignment rule: 1 = diagnosed with the chronic disease, 0 = not diagnosed with the chronic disease). For the 0/1 binary data, Yule's Q association metric was selected to transform the data into a distance matrix, which was used for subsequent cluster analysis. Hierarchical clustering was performed using the average-class method. This metric effectively captures both positive and negative co-occurrence relationships among the 10 chronic diseases while preserving irrelevant information features between variables, making it suitable for the binary co-occurrence data in this study. A dendrogram (phylogenetic tree) was generated through cluster analysis, and the final number of clusters was determined based on clinical significance to ensure the practical value and interpretability of the clustering results.

#### 2.3.4 Principal component analysis

PCA applies appropriate mathematical transformations to express new variables as linear combinations of original variables. It selects several principal components that account for a significant proportion of the total variance to analyze phenomena. In this study, a covariance matrix was constructed based on the 10 ADHD comorbidity variables. Through eigenvalue decomposition, it obtained the loading coefficients for each variable and the variance contribution rates of the principal components. The loading coefficients reflect the strength of association between comorbidities and principal components, while the variance contribution rates characterize the explanatory power of principal components over the original data.

## 3 Results

### 3.1 Basic information

A total of 52,098 children with ADHD were included in the study. Of these, 41,301 (79.3%) had only ADHD without any comorbidity, while 10,797 (20.7%) had at least one comorbid condition. In the overall sample, there were 10,974 (21.1%) females and 41,124 (78.9%) males, with a mean age of 8.1 ± 1.9 years. The majority (92.6%) were aged 6–11 years, and 7.3% were aged 12–17 years.

When comparing children with only ADHD and those with comorbidity, significant differences were observed in gender, age, and age group distribution (all *p* < 0.001). The proportion of females was higher in the comorbid group (25.2%) than in the only-ADHD group (20.0%). Children with comorbid ADHD were slightly older, with a mean age of 8.3 ± 2.1 years compared to 8.1 ± 1.9 years in the only-ADHD group. In terms of age group distribution, children aged 12–17 years accounted for a larger proportion in the comorbid group (9.8%) than in the only-ADHD group (6.7%) (Table 1).

**Table 1:** Demographic characteristics of children with ADHD: stratified analysis by comorbidity status.

Character	All *n* = 52,098	Only ADHD *n* = 41,301	With comorbidity *n* = 10,797	*P*
Sex				
Female *n* (%)	10,974 (21.1)	8,254 (20.0)	2,720 (25.2)	<0.001
Male *n* (%)	41,124 (78.9)	33,047 (80.0)	8,077 (74.8)	
Mean age ± SD	8.1 ± 1.9	8.1 ± 1.9	8.3 ± 2.1	<0.001
Age group (*Y*)				
6–11	48,268 (92.6)	38,526 (93.3)	9,742 (90.2)	<0.001
12–17	3830 (7.4)	2775 (6.7)	1,055 (9.8)	

Among children with ADHD, we identified and analyzed the 10 most common comorbid conditions, with prevalence rates ranging from 0.5 to 5.5%. The most frequent comorbidities were tic disorders (*n* = 2,845, 5.5%) and intellectual disabilities (*n* = 2,706, 5.2%), followed by ASD (*n* = 691, 1.3%), epilepsy (*n* = 507, 0.9%), obesity (*n* = 421, 0.8%), sleep problems (*n* = 397, 0.7%), learning disabilities (*n* = 358, 0.6%), mood disorders (*n* = 319, 0.6%), vitamin deficiencies (*n* = 267, 0.5%), and articulation disorders (*n* = 263, 0.5%).

Significant gender differences were observed for most comorbidities (except vitamin deficiencies, *p* = 0.386). Tic disorders, ASD, obesity, and articulation disorders were male-predominant, with male proportions ranging from 83.2 to 86.7% in the comorbid groups, compared to 78.7%−78.9% in children without these conditions. In contrast, intellectual disabilities, epilepsy, sleep problems, learning disabilities, and mood disorders showed higher female representation in the comorbid groups (female proportions 29.6%−43.9% vs. 20.5%−21.3% in non-comorbid groups).

Age differences were also significant for most comorbidities (except learning disabilities, *p* = 0.208, and vitamin deficiencies, *p* = 0.682). Children with mood disorders were the oldest (mean age 11.1 ± 2.1 years), followed by those with epilepsy and sleep problems (both 8.8 ± 2.3 years), obesity (8.6 ± 1.9 years), and tic disorders (8.3 ± 1.9 years). In contrast, children with ASD (7.2 ± 1.7 years), articulation disorders (7.3 ± 1.6 years), and intellectual disabilities (7.8 ± 1.9 years) were younger than those without these conditions (Supplementary Table 1).

### 3.2 Co-occurrence analysis

**Figure 2:**
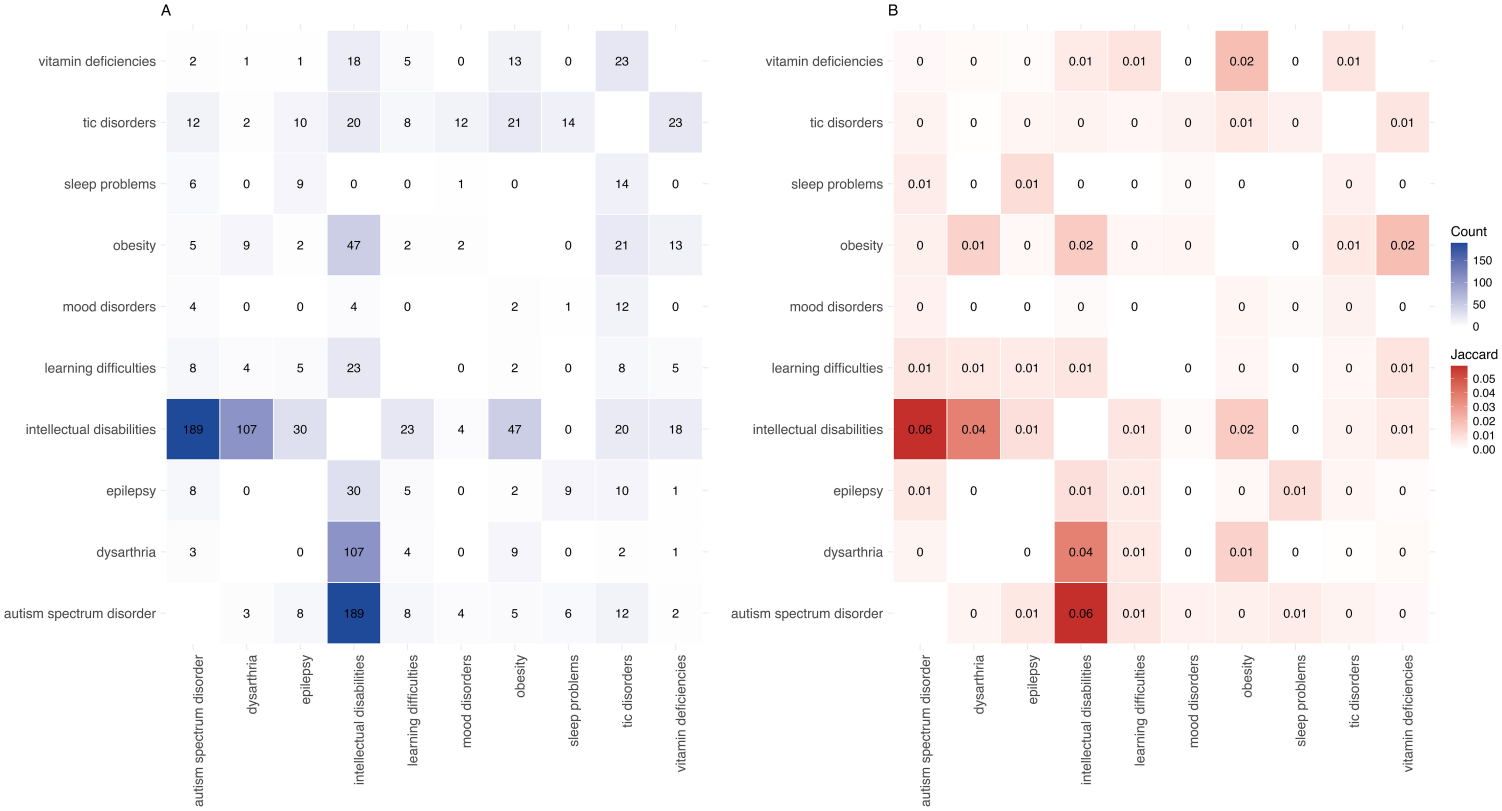
Co-occurrence of 10 chronic diseases or health problems. **(A)** Frequency of occurrence. **(B)** Jaccard similarity coefficients.

Among the 10,797 children with ADHD who had comorbid conditions, pairwise co-occurrence patterns of the 10 chronic diseases or health issues were analyzed. Figure 2A illustrates the frequency of pairwise disease co-occurrence. The most common comorbidity combinations were intellectual disability with ASD (*n* = 189) and intellectual disability with articulation disorder (*n* = 107).

Jaccard similarity coefficient analysis was performed to mitigate potential bias arising from differences in absolute disease frequencies. As shown in Figure 2B, the most prevalent comorbidity combination based on Jaccard similarity was ADHD with concurrent intellectual disability and ASD (Jaccard = 0.06), followed by intellectual disability with articulation disorder (Jaccard = 0.04).

**Figure 3:**
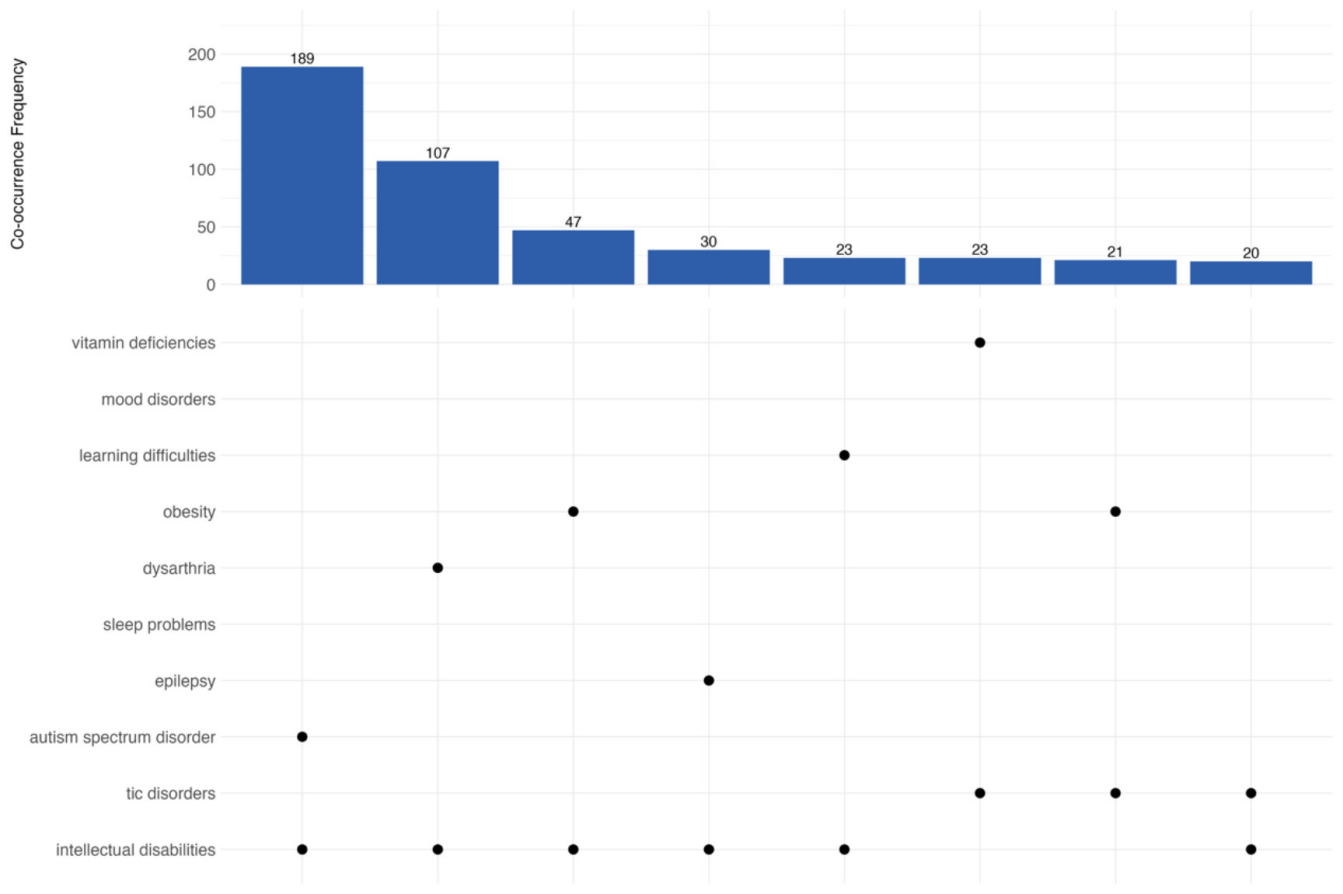
Frequency of the most comorbidity combinations and their co-occurrence in children with ADHD (frequency > 20).

Figure 3 presents the frequency of the most common comorbidity combinations among children with ADHD. Only combinations with an occurrence count greater than 20 are shown, and the sample size was 735 children with ADHD.

The most prevalent comorbidity pair was intellectual disability + ASD, observed in 189 children (25.7% of the sample size). The second most frequent combination was intellectual disability + dysarthria (107 cases, 14.5%). Additional common pairs included intellectual disability + obesity (47 cases, 6.4%), intellectual disability + epilepsy (30 cases, 4.0%), intellectual disability + learning difficulties (23 cases, 3.1%), tic disorder + vitamin deficiency (23 cases, 3.1%), tic disorder + obesity (21 cases, 2.8%), and intellectual disability + tic disorder (20 cases, 2.7%). Notably, intellectual disability was involved in six of the eight most frequent combinations, indicating a strong tendency for this condition to co-occur with other neurodevelopmental, neurological, and metabolic disorders in children with ADHD. Tic disorders also demonstrated a clear pattern of co-occurrence, appearing in three distinct combinations.

### 3.3 Cluster analysis

**Figure 4:**
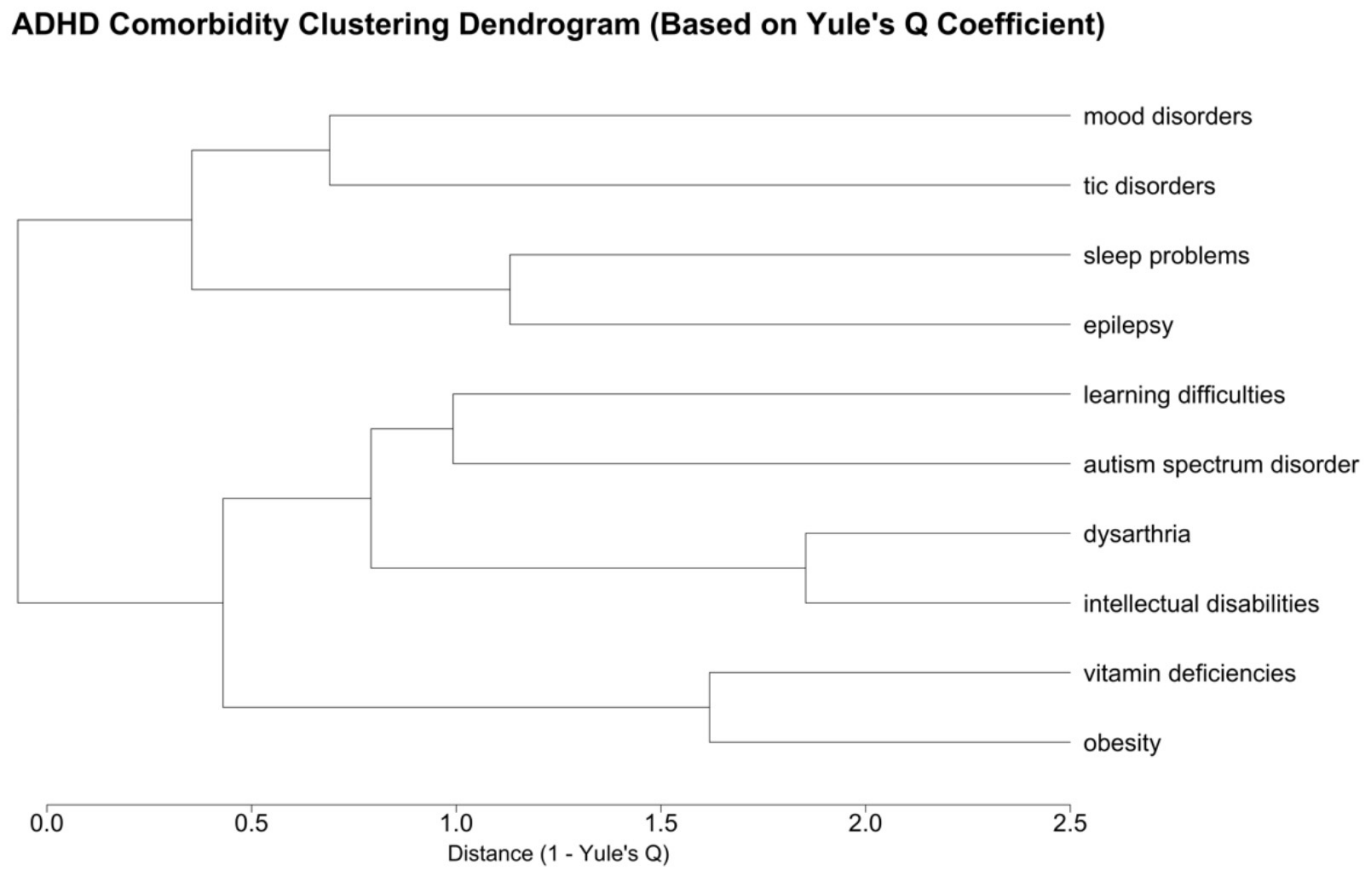
Dendrogram of systematic clustering of 10 common chronic conditions or health problem.

Yule's Q distance reflects the prevalence patterns of comorbidities within the study population, where closer distances indicate higher relative co-occurrence rates and greater shared risk factors. At the lowest distance thresholds, mood disorders and tic disorders formed the closest pair, followed by learning difficulties and ASD, sleep problems and epilepsy, dysarthria and intellectual disabilities, and vitamin deficiencies and obesity. At higher hierarchical levels, these pairs aggregated into two major clusters. The first cluster comprised mood disorders, tic disorders, sleep problems, and epilepsy. The second cluster included two subclusters: one consisting of learning difficulties, ASD, dysarthria, and intellectual disabilities, and the other consisting of vitamin deficiencies and obesity. These findings demonstrate that ADHD comorbidities are not randomly distributed but form distinct, functionally related clusters, with stronger associations observed among neurodevelopmental and neuropsychiatric conditions compared to metabolic comorbidities (Figure 4).

### 3.4 PCA

PCA was performed to further identify the core comorbidity patterns of ADHD. Five principal components were selected based on the inflection points in the scatter plot, cumulative variance contribution rates, and combined with clinical research requirements. Collectively, these five principal components accounted for 56.6% of the cumulative variance, which effectively covered the core characteristics of ADHD comorbidities. A loading coefficient > 0.36 was defined as the screening criterion for core comorbidities, aiming to identify diseases with a strong association with the principal components. For comorbidities with a loading coefficient ≤ 0.36, the corresponding principal component was assigned based on the principle of maximum loading, which served as supplementary weakly associated diseases. All principal components were mutually exclusive to avoid overlapping interpretations. The results demonstrated that the first principal component was represented by tic disorders, the second by intellectual disabilities, the third by epilepsy, the fourth by sleep problems, and the fifth by obesity. Detailed information on the principal component classification is presented in Table 2.

**Table 2:** Principal component analysis (PCA) of comorbidity patterns in children with ADHD.

Cluster group	Comorbidity	RC1	RC2	RC3	RC4	RC5
Cluster1	Tic disorders	0.82	–	–	–	–
	Mood disorders	0.18	–	–	–	–
Cluster2	Intellectual disabilities	–	0.79	–	–	–
	Dysarthria	–	0.20	–	–	–
	Learning difficulties	–	0.28	–	–	–
	Autism spectrum disorder	–	0.25	–	–	–
Cluster3	Epilepsy	–	–	0.81	–	–
Cluster4	Sleep problems	–	–	–	0.80	–
Cluster5	Obesity	–	–	–	–	0.78
	Vitamin deficiencies	–	–	–	–	0.15

## 4 Discussion

This study collected clinical data from pediatric patients to characterize the comorbidity patterns of ADHD. Benefiting from the standardized assessment tools adopted in this study, including CBCL, SNAP-IV and WFIRS, which are all clinically validated and reliable scales, as well as the structured clinical interviews based on the DISC-IV conducted by professional physicians, we could accurately collect and quantify the relevant data. We further explored the combination patterns and clustering characteristics of multiple comorbidities among children with ADHD. The findings supplement epidemiological data on multimorbidity in ADHD populations and enrich baseline demographic data in this field, providing evidence for clinicians to facilitate early identification and timely screening of potential comorbid conditions in children with ADHD. We observed that ADHD onset predominantly occurred between the ages of 6 and 11 years, which is consistent with the findings of previous research (7). Approximately 21% of children with ADHD exhibited comorbid conditions. This prevalence rate appears lower than that reported in earlier studies (8, 9), potentially attributed to methodological and clinical factors. First, assessments relying on parental reports via CBCL, SNAP-IV and WFIRS may be influenced by caregivers' educational background, cognitive level and emotional state. Some parents might conceal or downplay children's mild symptoms due to stigma concerns, resulting in the omission of subtle behavioral manifestations. Second, clinicians have relatively insufficient awareness and recognition of ADHD comorbidity in routine clinical practice, which may lead to underdiagnosis of comorbid conditions. Furthermore, the DSM-5 criterion extending the age of symptom onset from before 7 years to before 12 years has broadened the diagnostic spectrum and changed the demographic composition of affected children, which may also account for the between-study difference in comorbidity prevalence. Our findings indicate that boys are approximately three times more likely than girls to develop ADHD, which aligns with global prevalence trends (7, 10). Some studies have suggested that females may have a protective effect against developing ADHD (11). Additionally, some scholars have contended that ADHD in females is significantly underdiagnosed. Unlike males, who typically exhibit hyperactivity/impulsivity alongside inattention, females may present solely with attention-related difficulties without pronounced hyperactivity—a clinical feature that potentially leads to missed diagnoses (12). Furthermore, we identified associations between gender, age, and comorbid conditions. However, we cannot determine whether these associations are unique to ADHD or merely replicate patterns of comorbidity prevalence differences across genders and ages observed in the general population.

Tic disorders were the most frequently detected comorbidity among the ADHD population in this study, with a high co-occurrence rate that is consistent with existing research findings (13). This consistent observation suggests a close pathophysiological association between ADHD and tic disorders. From a pathophysiological perspective, both ADHD and tic disorders involve dysfunction in the basal ganglia-cortex-thalamus circuit. Impairment in this circuit, which plays a core role in regulating motor control and impulsive behavior, coupled with imbalances in the secretion and metabolism of neurotransmitters such as dopamine and serotonin, constitutes the key neurobiological basis for their comorbidity (13). Additionally, family genetic studies have revealed partially overlapping genetic susceptibility genes between the two disorders, which further clarifies the genetic underpinnings of their co-occurrence (14). Therefore, in clinical diagnosis and treatment, symptoms of both conditions must be comprehensively considered to avoid suboptimal therapeutic outcomes resulting from single-disorder-focused interventions.

Intellectual disability was the second most frequently detected comorbidity among children with ADHD in this study. Research indicates that the prevalence of ADHD co-occurring with intellectual disability reaches 14%, compared to 1% in the general population (15). A study investigating the subsequent development of ADHD in children with intellectual disability suggested that these children were more likely than their typically developing peers to develop the hyperactive/impulsive type of ADHD (RR = 4.43; 95% CI [2.32, 8.44]) (16). However, not all children with intellectual disability who exhibit high levels of hyperactivity symptoms meet the diagnostic criteria for ADHD. This is primarily because their intellectual development lags behind that of typically developing peers without intellectual disability, which may lead to overlapping behavioral manifestations that are not necessarily indicative of comorbid ADHD. The DSM-5 specifies that a dual diagnosis of ADHD and intellectual disability is appropriate in children with intellectual disabilities, provided that developmental functioning is taken into account during the diagnostic process. Nevertheless, research findings regarding the co-occurrence of intellectual disability and ADHD remain controversial (17–19). Therefore, clinicians should exercise caution when evaluating and diagnosing ADHD symptoms in children with intellectual disabilities to avoid misdiagnosis.

ASD and ADHD both fall under the category of neurodevelopmental disorders. The co-occurrence of these two conditions observed in our study is consistent with the comorbidity patterns commonly seen among neurodevelopmental disorders. In clinical practice, distinguishing between ASD and ADHD can be challenging. Prior to the release of DSM-5, clinicians were unable to diagnose ADHD in patients with ASD. It was previously believed that any symptoms of inattention and/or hyperactivity-impulsivity were merely secondary manifestations of ASD. With the removal of this exclusionary criterion in DSM-5, a substantial body of literature focusing on the comorbidity of ADHD and ASD has emerged in recent years (20, 21). Multiple large-scale ADHD cohort studies have reported that approximately 12% of ADHD cases co-occur with ASD (8, 22). Both ASD and ADHD are highly heritable neurodevelopmental disorders; however, the specific pathophysiology and molecular mechanisms underlying their comorbidity remain unclear.

Our data indicate that among all combinations of comorbid conditions in children with ADHD, the most prevalent pairing is intellectual disability and ASD. This finding is consistent with previous studies, which have also identified ADHD, ASD, and intellectual disability as common comorbid disorders (15). Notably, one in every two adolescents with both intellectual disability and ASD exhibits clinically significant inattention, hyperactivity, and/or impulsive behaviors (23). Additionally, Holly K. et al. identified potential harmful variants in RFX3, RFX4, and RFX7 genes across patients with intellectual disability, ADHD, and ASD, providing preliminary insights into the genetic basis of their comorbidity (24).

The comorbidity of epilepsy and ADHD reflects an interplay between abnormal electrical activity in the central nervous system and defects in functional regulation. The prevalence of ADHD reaches as high as 77% among patients with refractory epilepsy, compared to 3%−5% in the general population. Prenatal exposure to antiepileptic drugs and shared genetic backgrounds suggest that this comorbidity is far from coincidental (25). This underscores the need for routine electroencephalogram (EEG) monitoring in ADHD patients to promptly detect potential epileptic seizures and prevent further brain dysfunction.

In this study, the number of ADHD cases co-occurring with obesity ranked among the highest. Similarly, a meta-analysis revealed that children with ADHD face an increased risk of overweight and obesity (OR = 1.56; 95% CI [1.32–1.85]), particularly among boys (OR = 1.45; 95% CI [1.10–1.90]) (26). Inattentiveness may lead to obesity by reducing an individual's sensitivity to internal satiety signals (27). Although the number of detected cases of vitamin deficiency was relatively low—consistent with previous literature findings (28, 29) —it still suggests an association between nutritional metabolic status and ADHD. This indicates that in clinical interventions for ADHD patients, attention should be paid not only to neurodevelopmental symptoms but also to dietary and nutritional management. Specifically, interventions should include adjusting the dietary structure, supplementing deficient vitamins, and regulating body weight.

Although sleep problems were detected in a low number of cases among ADHD comorbidities in this study, they still warrant clinical attention. Sleep deprivation often exacerbates the core symptoms of ADHD, while the impulsivity and hyperactivity symptoms of ADHD further disrupt sleep patterns, thereby creating a vicious cycle. A Swedish study found that 7.5% of ADHD patients were diagnosed with sleep problems, and 47.5% had been prescribed sleep medications (30). These findings suggest that improving sleep quality may emerge as a critical target for comprehensive ADHD interventions.

To further explore the comorbidity patterns of ADHD, we employed complementary cluster analysis and PCA. In the cluster analysis, the association between intellectual disability, ASD, as well as articulation disorders and learning disabilities, reflects the profound impact of neurodevelopmental abnormalities on learning and language abilities. Notably, the clustering of obesity and vitamin deficiencies challenges the traditional understanding that ADHD comorbidities predominantly cluster within the neurodevelopmental domain, suggesting that the ADHD comorbidity spectrum also encompasses somatic metabolic and nutritional status-related conditions. Sleep problems and epilepsy are both closely associated with abnormal excitability in the central nervous system, which reflects potential pathological features of abnormal brain electrical activity regulation in ADHD patients. Tic disorders and mood disorders both involve abnormalities in neurotransmitter synthesis and metabolism, indicating shared neurobiological underpinnings between these two comorbidities. PCA reduced dimensionality to extract five core components from multiple comorbidities: tic disorders, intellectual disabilities, epilepsy, sleep problems, and obesity. This outcome clarifies the core manifestations of ADHD comorbidities and provides a simplified yet comprehensive overview of their distribution patterns. Collectively, these research methods provide key indicators for clinical screening, condition assessment, and prognosis evaluation of ADHD comorbidities.

This study has several notable strengths. It adopted a large sample size and spanned a long observational period, which improves the stability and representativeness of the results. It systematically explored and summarized the patterns of the most common comorbidities among children with ADHD, further clarifying the demographic characteristics and distribution differences of various comorbid conditions. Nevertheless, several limitations of the present study should be acknowledged. First, this study was conducted at a single center, which may bring inevitable selection bias to the enrollment of participants. Second, this study adopted a retrospective design. Due to the constraints of retrospective data collection, we were unable to obtain information on the ADHD subtypes of the included children, thus preventing further exploration of the potential association between different ADHD subtypes and comorbid condition. Additionally, influenced by clinicians' awareness of ADHD comorbidities, some conditions may have been overlooked or incompletely documented during clinical care, potentially affecting the completeness of our findings. Moving forward, we aim to conduct a multicenter, longitudinal study on ADHD comorbidities. Such a study design will help address the current limitations and provide more scientific, reliable, and generalizable clinical evidence to support personalized and comprehensive interventions for children with ADHD.

## 5 Conclusion

This study characterizes the prevalence of common comorbidities in pediatric ADHD and reveals the burden that these comorbid conditions impose on affected children. By elucidating the heterogeneity and core characteristics of comorbidities, it provides crucial data support and new insights for subsequent precision assessment, individualized interventions, and mechanism research. Clinicians are therefore encouraged to develop targeted intervention plans following a comprehensive assessment of each child's condition, addressing neurodevelopmental, physical health, and emotional wellbeing to improve overall outcomes for ADHD patients.

## Data Availability

The raw data supporting the conclusions of this article will be made available by the authors, without undue reservation.
