# Supplementary material for: Epidemiological characteristics of comorbidities in childhood Attention-Deficit/Hyperactivity Disorder and common comorbidity patterns
Source: Front Public Health. 2026 Jul 21;14:1838844. doi: 10.3389/fpubh.2026.1838844 (PMC13435844; doi:10.3389/fpubh.2026.1838844)
Supplement: Supplementary file 1 [file Table_1.docx]

| Comorbidity | Group | N (%) | Female n (%) | Male n (%) | *P* (gender) | Mean age ± SD | *P*  (age) |
| --- | --- | --- | --- | --- | --- | --- | --- |
| Tic disorders | ADHD with tic disorders | 2,845 (5.5) | 477 (16.8) | 2,368 (83.2) | <0.001 | 8.3 ± 1.9 | <0.001 |
|  | ADHD without tic disorders | 49,253 (94.5) | 10,497 (21.3) | 38,756 (78.7) |  | 8.1 ± 1.9 |  |
| Intellectual disabilities | ADHD with Intellectual disabilities | 2,706 (5.2) | 858 (31.7) | 1,848 (68.3) | <0.001 | 7.8 ± 1.9 | <0.001 |
|  | ADHD without Intellectual disabilities | 49,392 (94.8) | 10,116 (20.5) | 39,276 (79.5) |  | 8.1 ± 1.9 |  |
| Autism spectrum disorder | ADHD with autism spectrum disorder | 691 (1.3) | 98 (14.2) | 593 (85.8) | <0.001 | 7.2 ± 1.7 | <0.001 |
|  | ADHD without autism spectrum disorder | 51,407 (98.7) | 10,876 (21.2) | 40,531 (78.8) |  | 8.1 ± 1.9 |  |
| Epilepsy | ADHD with epilepsy | 507 (0.9) | 183 (36.1) | 324 (63.9) | <0.001 | 8.8 ± 2.3 | <0.001 |
|  | ADHD without epilepsy | 51,591 (99.1) | 10,791 (20.9) | 40,800 (79.1) |  | 8.1 ± 1.9 |  |
| Obesity | ADHD with obesity | 421 (0.8) | 62 (14.7) | 359 (85.3) | 0.001 | 8.6 ± 1.9 | <0.001 |
|  | ADHD without obesity | 51,677 (99.2) | 10,912 (21.1) | 40,765 (78.9) |  | 8.1 ± 1.9 |  |
| Sleep problems | ADHD with sleep problems | 397 (0.7) | 124 (31.2) | 273 (68.8) | <0.001 | 8.8 ± 2.3 | <0.001 |
|  | ADHD without sleep problems | 51,701 (99.3) | 10,850 (21.0) | 40,851 (79.0) |  | 8.1 ± 1.9 |  |
| Learning disabilities | ADHD with learning disabilities | 358 (0.6) | 106 (29.6) | 252 (70.4) | <0.001 | 8.2 ± 1.9 | 0.208 |
|  | ADHD without learning disabilities | 51,740 (99.4) | 10,868 (21.0) | 40,872 (79.0) |  | 8.1 ± 1.9 |  |
| Mood disorders | ADHD with mood disorders | 319 (0.6) | 140 (43.9) | 179 (56.1) | <0.001 | 11.1 ± 2.1 | <0.001 |
|  | ADHD without mood disorders | 51,779 (99.4) | 10,834 (20.9) | 40,945 (79.1) |  | 8.1 ± 1.9 |  |
| Vitamin deficiencies | ADHD with vitamin deficiencies | 267 (0.5) | 62 (23.2) | 205 (76.8) | 0.386 | 8.0 ± 1.8 | 0.682 |
|  | ADHD without vitamin deficiencies | 51,831 (99.5) | 10,912 (21.1) | 40,919 (78.9) |  | 8.1 ± 1.9 |  |
| Articulation disorders | ADHD with articulation disorders | 263 (0.5) | 35 (13.3) | 228 (86.7) | 0.002 | 7.3 ± 1.6 | <0.001 |
|  | ADHD without articulation disorders | 51,835 (99.5) | 10,939 (21.2) | 40,896 (78.9) |  | 8.1 ± 1.9 |  |

Supplementary Table 1 Demographic characteristics of children with ADHD, stratified by the presence of the 10 most common comorbidities
